# Antibiotics prescription and guidelines adherence in elderly: impact of the comorbidities

**DOI:** 10.1186/s12877-019-1265-1

**Published:** 2019-10-29

**Authors:** Anthony Dylis, Anne Sophie Boureau, Audrey Coutant, Eric Batard, François Javaudin, Gilles Berrut, Laure de Decker, Guillaume Chapelet

**Affiliations:** 1 Clinical Gerontology Department, Centre Hospitalier Universitaire de Nantes, 1 place Alexis-Ricordeau, F-44000 Nantes, France; 2 Université de Nantes, EE MiHAR (Microbiotes, Hôtes, Antibiotiques et Résistance bacterienne), Institut de Recherche en Santé (IRS2), 22 Boulevard bénoni-Goullin, F-44200 Nantes, France; 3 Emergency Department, Centre Hospitalier Universitaire de Nantes, 1 place Alexis-Ricordeau, F-44000 Nantes, France

**Keywords:** Aged, 80 and over, Antibacterial agent, Charlson score comorbidity, Inappropriate prescribing

## Abstract

**Background:**

Although the interest of antibiotics is well known, antibiotics prescription is associated with side effect, especially in patients with multiples comorbidities. One way to reduce the incidence of side effects is to respect antibiotics prescriptions guidelines. Our objective was to investigated the factors associated with guidelines adherence in elderly patients with multiples comorbidities.

**Methods:**

From October 2015 to December 2016, antibiotics prescription and guidelines adherence were analyzed in two post-acute care and rehabilitation services of a 2600-bed, university-affiliated center.

**Results:**

One hundred and twenty-eight patients were included, fifty-nine (46%) patients had antibiotics prescription according to guidelines. In Multivariable logistic regression analysis, prescription of 2 antibiotics or more (OR = 0.168, 95% IC = 0.037–0.758, *p* < 0.05), 85 years of age and more (OR = 0.375, 95% IC = 0.151–0.931, *p* < 0.05) and the Charlson comorbidity index score (OR = 0.750, 95% IC = 0.572–0.984, *p* < 0.05) were negatively associated with antibiotics prescriptions according to guidelines.

**Conclusions:**

High comorbidity in the elderly was negatively associated with the guidelines adherence of antibiotiсs prescriptions. These criteria should be considered to optimize antibiotics prescriptions in elderly patients.

**Electronic supplementary material:**

The online version of this article (10.1186/s12877-019-1265-1) contains supplementary material, which is available to authorized users.

## Introduction

Antibiotics are among the most prescribed drugs in the world [1]. Since the 2000s, there has been an increase of more than 20% of antibiotics prescriptions in elderly patients [2]. Antibiotics have undeniable benefit effects, but have also side effects that could represent a serious threat to public health [3]. Good quality of antibiotics prescription, defined by an adapted use of an antibacterial agent during an infection (molecule, dose and duration) and by the adherence of prescription guidelines, is associated with less side-effect [4, 5]. Previous studies found that between 25 and 75% of antibiotics prescriptions did not meet the guidelines [6, 7]. To our knowledge, no studies have examined the relationship between the number of comorbidities and the adherence of antibiotics prescriptions guidelines in elderly patients [8], and considering the others confounding factors associated with antibiotic prescription [9–13].

The morbidity burden is defined by Valderas et al. as the total burden of types of illness having an impact on an individual’s physiological reserve [14]. The presence of a high burden of comorbidity increases early mortality in bacterial infection, the number of hospitalizations and has a high public health impact [15]. A recent study in primary care found that a high burden of comorbidities is associated with an increased in antibiotic initiation [16]. However, this study did not specially examine whether treatments were initiated for bacterial infection and whether they were prescribed according to guidelines.

The objective of this study was to examine the relationship between a high comorbidity index score and the adherence to antibiotics prescriptions guidelines, in elderly patients.

## Methods

### Study population

This retrospective cohort study was conducted in 2017 in Nantes University Hospital, a 2600-bed, university-affiliated center, in the Clinical Gerontology Department, Nantes, France. All patients hospitalized in the post-acute care and rehabilitation centers during 1st October 2015 and 31 December 2016 were screened before inclusion. Patient who received antibiotics treatments (enteral and parenteral) were identified and their electronic medical record were reviewed by two independent investigators. If the necessary information were not available in the electronic medical records, the medical papers records were reviewed. For inclusion, antibiotics had to be prescribed for suspicion of bacterial infection.

Bacterial infection was characterized according to the Mac Geer criteria [17]. These criteria take into account clinical and paraclinical presentation. Infection was considered as certain if all clinical and paraclinical criteria were presents. Infection was considered as probable if only clinical criteria were presents. For example, certain or probable case of pulmonary infection was distinguished by the presence of radiological pneumonia. If no criterion was present, antibiotic prescription was considered as not justified. A patient was not included if antibiotic was initiated in another service, prescribed for prophylaxis, or by local application. Only the first antibiotic prescription was taken into consideration.

### Adherence with guidelines

The main study end-point of this study was adherence with guidelines. According to previous studies [6, 9], antibiotics prescriptions were considered in adherence if all the following criteria met the guidelines: molecule, dose and duration. The Guidelines (Additional file 1: Table S1) provided by the French Infectious Disease Society were considered [18–21]. Patients with other source of infection were not included. Antibiotics prescriptions recommendations are similar than those proposed by international guidelines [22–24].

Finally, patients were divided into two groups considering groups according to the presence or absence of adherence to the recommendations.

### Charlson comorbidity index score and confounding factors

The Charlson comorbidity index score was used for the evaluation of the burden of comorbidities [25]. According to previous studies, the following variable were collected because of their associations with guidelines adherence or inappropriate drugs prescription in elderly patients [9–13]: 85 years and older, gender, cognitive impairment, history of falls during the last month, obesity (Body Mass Index > 30), presence of an indwelling urinary catheter, peritonitis, joint or bone infection, macrolide prescription, carbapenem prescription, first generation cephalosporin prescription, prescription of 2 antibiotic or more, 10 or more drugs per day, Instrumental of Activities Daily Living score (IADL), Activities of Daily Living score (ADL); serum creatinine higher than 120 μmol/L, recent advice from an infectiologist.

### Statistical analysis

The participant’s baseline characteristics were summarized using mean and standard deviations or frequencies and percentages, as appropriate. Between-group comparisons were performed using an independent simple T-test or chi square test (X^2^) as appropriate. Univariable and Multivariable logistic regressions were performed to examine association between antibiotic prescription according to the guidelines and others variables. Variables with a significant association in Univariable analysis and/or with *p*-value < 0.2 were entered into a logistic regressions model for Multivariable analysis. Relative risks were expressed as odds ratios (OR) and 95% confidence intervals. All reported p-value < 0.05 were considered as statistically significant. Analysis was performed using SPSS software version 15.0 (SPSS, Inc., Chicago, IL, USA).

### Ethical consideration

The study was conducted in accordance with the ethical standards set forth in Helsinki declaration (1983). The local ethical committee approved the study protocol.

## Results

### Patient characteristics

**Fig. 1:**
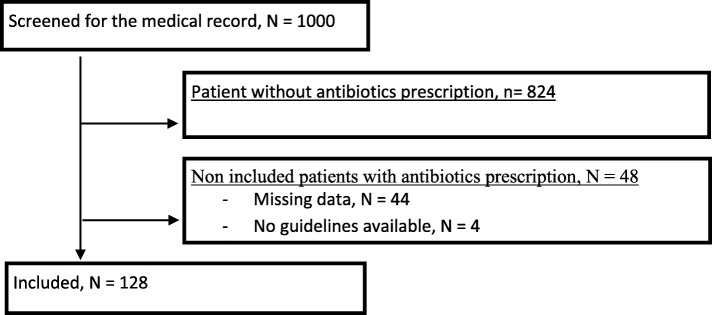
Screening, exclusion and enrollment process of participants, *N* = 128

A thousand files were reviewed before inclusion (Fig. 1). Among them, 824 patients did not have antibiotics prescription. Among the 176 remaining patients, 48 were excluded: 4 because no guidelines could be used to assess the primary outcomes, 44 because no clinical or paraclinical information were available. Finally, 128 patients were included. The characteristics of the population are shown in Table 1 and Additional file 1: Table S2: 93 (73%) patients were women, median age were 87 years old, the mean Charlson’s comorbidity was 2.48.

**Table 1:** Demographic and clinical characteristics of total population, *N* = 128

	Total *N* = 128 (%)	Antibiotics prescription in accordance with the guidelines *N* = 59 (%)	Antibiotics prescription not in accordance with the guidelines *N* = 69 (%)	*p*-Value *
Age. Years, mean +/−SD	87.05 (5.402)	85.69 (5.593)	88.22 (4.985)	0.008
85 years and more, n (%)	85 (66.4)	33 (55.9)	52 (75.4)	0.025
Women, n (%)	93 (72.7)	47 (79.7)	46 (66.7)	0.115
Length Of Stay (day) +/− SD	48.79 (26.145)	51.32 (25.741)	46.31 (26.523)	0.307
Charlson score +/− SD	2.48 (2.280)	2.17 (2.127)	2.75 (2.385)	0.149
ADL +/− SD	4.70 (1.297)	4.80 (1.275)	4.62 (1.319)	0.440
IADL +/− SD	2.35 (1.390)	2.38 (1.437)	2.32 (1.359)	0.819
Serum albumin (g/L) +/− SD	30.87 (4.486)	31.11 (4.897)	30.66 (4.111)	0.576
Antibiotics treatment duration (day) +/− SD	7.80 (3.067)	7.70 (1.832)	7.88 (3.851)	0.754
≥ 2 antibiotics, n (%)	17 (13.3)	3 (5.1)	14 (20.3)	0.017
History of fall in the last month, n (%)	41 (32)	20 (33.9)	21 (30.4)	0.707
Chronic obstruction pulmonary disease, n (%)	6 (4.7)	2 (3.4)	4 (5.8)	0.686
Cognitive impairment, n (%)	29 (22.7)	16 (27.1)	13 (18.8)	0.295
Infectious advice, n (%)	2 (1.6)	1 (1.7)	1 (1.4)	1.000
Incontinence, n (%)	19 (14.8)	8 (13.6)	11 (15.9)	0.805
BMI > 30 kg/m^2^, n (%)	14 (10.9)	6 (10.2)	8 (11.6)	1.000
Presence of urinary catheter, n (%)	14 (10.9)	3 (5.1)	11 (15.9)	0.086
History of antibiotic allergy, n (%)	11 (8.6)	4 (6.8)	7 (10.1)	0.545
Presence of pressure ulcer, n (%)	9 (7)	6 (10.2)	3 (4.3)	0.300
Palliative care, n (%)	14 (10.9)	4 (6.8)	10 (14.5)	0.256
Over 10 treatments, n (%)	16 (12.5)	10 (16.9)	6 (8.7)	0.187
Serum creatinine > 120 μmol/L, n (%)	18 (14.1)	7 (11.9)	11 (15.9)	0.612

### Antibiotics prescriptions characteristics

Antibiotics were prescribed for the following infectious disease: 32 (25%) for pneumonia, 37 (29%) for cystitis, 11 (9%) for upper urinary tract infection (women) and 11 (9%) for male urinary tract infection (Additional file 1: Table S2). Among the 128 patients, guidelines were respected in 59 (46%) patients, 32 (25%) patients received antibiotics for uncertain diagnosis and 96 (75%) received antibiotics for certain or probable bacterial infection. Among these 96 patients, 87 (90%) patients had an adapted molecule (Additional file 1: Table S1), 62 (71%) patients had an adapted molecule and an appropriate duration and 59 (61%) patients had molecule, duration and dose adapted.

### Factors influencing antibiotic prescription according to the guidelines

In Multivariable analysis (Table 2), the following factors were significantly associated with antibiotic prescription in accordance with guidelines: prescription of 2 antibiotics or more (OR = 0.168, 95% IC = 0.037–0.758, *p* < 0.05); 85 years of age and more (OR = 0.375, 95% IC = 0.151–0.931, *p* < 0.05); Charlson comorbidity index score (OR = 0.750, 95% IC = 0.572–0.984, *p* < 0.05).

**Table 2:** Univariable and Multivariable logistic regression analysis of factors associated with antibiotic prescriptions according to the guidelines

	Univariable Analysis		Multivariable Analysis	
	OR (95% CI)	*p*-Value	OR (95% CI)	*p*-Value
85 years and more	0.415 (0.196–0.879)	0.022	0.375 (0.151–0.931)	0.035
Women	1.958 (0.873–4.392)	0.103	0.897 (0.311–2.585)	0.840
Charlson score	0.899 (0.757–1.044)	0.152	0.750 (0.572–0.984)	0.038
ADL	1.118 (0.844–1.482)	0.436	1.008 (0.659–1.543)	0.969
IADL	1.031 (0.795–1.337)	0.817	1.047 (0.7–1.566)	0.824
cognitive impairment	1.603 (0.697–3.686)	0.267	1.217 (0.442–3.354)	0.704
Infectious advice	1.17 (0.07–19.16)	0.911	0.852 (0.044–16.629)	0.916
BMI > 30	0.863 (0.281–2.647)	0.797	0.729 (0.162–3.286)	0.681
Presence of urinary catheter	0.282 (0.075–1.066)	0.062	0.224 (0.041.245)	0.087
Palliative care	0.429 (0.127–1.448)	0.173	3.084 (0.450–21.128)	0.251
Over > 10 therapy	2.143 (0.729–6.302)	0.166	3.510 (0.765–16.096)	0.106
Serum creatinine > 120 μmol/L	0.698 (0.252–1.933)	0.489	1.839 (0.488–6.933)	0.368
≥ 2 antibiotics	0.210 (0.057–0.773)	0.19	0.168 (0.037–0.758)	0.02

## Discussion

In this study, the following variables were negatively associated with antibiotic prescription according to the guidelines: Charlson’s comorbidity index score, prescription of 2 antibiotics, patients aged 85 years and older.

The prevalence of antibiotics prescriptions in adhrence with guidelines (46%) is consistent with other studies but is higher than another study carried out in a French nursing home [6, 26]. Indeed, Boivin et al [6] have found a prevalence of 17%. We make the assumption that this lower prevalence could be explained by three main arguments. First, quinolones prescriptions were considered as inappropriate by Boivin et al, and appropriate in this study, according to the guidelines [18, 19]. Second, we did not consider if antibiotics prescriptions were reevaluated and switched at 48-72 h because of a high number of missing data. Third we assume that the MacGeer criteria could improve the accuracy of the diagnosis and could increase the number of appropriate prescriptions.

In this study, a high Charlson’s comorbidity index score was a risk factor of antibiotics prescriptions not in adherence with guidelines. To our knowledge, this is the first description of this association. Previous studies have examined the association between antibiotics initiation and presence of comorbidities [27, 28], but without considering if the prescription was in adherence with guidelines. We assume that, together, two antibiotics bad practices could explain this result: inappropriate antibiotic initiation and absence of antibiotics prescription reevaluation. First, a physician could prescribe antibiotics earlier, and inappropriately in order to anticipate decompensation of comorbidities related to sepsis [28]. Second, Boivin et al. have found that a higher burden of comorbidities is associated with an absence of a systematic antibiotic prescription reevaluation [6]. Moreover, the study was conducted in a clinical ward where patients are treated by different senior doctors. Thus, prescribers were not always the one who reevaluated and stopped the prescription. We assume that this organization could also explain the difficulty to reevaluate and/or to stop the antibiotic.

In this study, 85 years of age and more has been negatively associated with antibiotic prescription in accordance with guidelines. To our knowledge, this is the first description of this association, that was previously described in another study that analyzed factors associated with inappropriate prescriptions in the elderly [11]. We assume that this result could be explained by two main arguments. First, age could have an impact on an earlier initiation of the antibiotics prescription. Indeed, Brooke et al. [29], in a study about factors influencing antibiotics prescription, proposed that physician may have fear of a rapid clinical aggravation in elderly patients, even in absence of clear evidence of bacterial infection. Second, as it was proposed for the Charlson comorbidity index score, the physician could have fear to stop antibiotics prescriptions in elderly patients even in absence of a high burden of comorbidities.

In this study, prescription of two antibiotics has been negatively associated with antibiotics prescription according to the guidelines. Kawanami et al. have already found this association in a recent study [13]. Our study confirms this result in a more specific population, vulnerable to side effects associated with antibiotics prescription [5]. This result can be explained by two arguments. First, physicians could initiate broad spectrum antibiotics prescriptions considering that the diagnosis of site of infection is not certain because of atypical clinical presentations [4]. Then, for the same reason, deprescribing to a narrower spectrum antibiotic may not be done [30]. In our study, no patient who initially received ceftriaxone or imidazole had further deprescription to a narrower-spectrum antibiotic. Second, we assume that a switch for another antibiotic can lead to inappropriate prescriptions. In this study, only three prescriptions were in accordance to the guideline when the antibiotic was switched for a narrower spectrum molecule, with a longer treatment duration.

Although it was previously described [9, 13], we did not find an association between guidelines adherence and the site of infection or the administration of a parenteral antimicrobial treatment. We assume that two methodological biases could explain these results. First our study may have been underpowered to detect these associations. Second, the population characteristics could be different. Indeed, Kawanami et al. included hospitalized patient, without age limit and described more gastro-intestinal infections, peritoneum infections or bone infections, associated with an inappropriate antibiotic prescription [9, 13]. Moreover, in our study, no patient has been treated with cephalosporin 1st generation or aminoglycoside, which are antibiotics associated with inappropriate prescription [9, 13].

This study has limitations. First, this study had an observational design and we report the result of a retrospective analysis. Therefore, hidden bias could exist and may have underestimated the relationship between some factors and guidelines adherence. Second, because of the retrospective design, it was difficult to collect social or economic or morbimortality parameters that could be associated with guidelines adherence. Indeed, this study was not designed to analyze whether guidelines adherence is cost effective or reduced morbidity and mortality. Moreover, guidelines sometimes ignore vulnerable population such as frail elderly patients. Third, it is a French monocentric and study and the results may not be fully applicable to others settings with others guidelines. However, we assume that this population is representative of a population particularly vulnerable to infectious disease and with a higher risk of side-effects associated with antibiotics prescriptions [5]. Indeed, patients were predominantly female, with a median age of 87 years old, required assistance for activities of daily living, and the mean Charlson’s index score was 2.48. Fourth, the Mac Geer criteria may not be fully adapted for the accuracy of the diagnosis of infection. We used the Mac Geer criteria because they take into account atypical semiological presentation in elderly patients [17], and we assume that they can be useful to asses antibiotics prescription in this population.

## Conclusion

This study highlights the importance of co-morbidities in the prescription of antibiotics and more particularly on compliance with the guidelines. Further research is needed to identify other parameters involved in adherence with guidelines. Considering the impact on public health, these factors should be integrated into research protocols to improve and optimize antibiotic prescriptions.

## Additional file

Additional file 1: **Table S1.** Site of infection and antibiotics recommended by the prescriptions guidelines. **Table S2.** Clinical characteristics of total population: site of infection and Antibiotics prescribed. (DOCX 28 kb)

## Data Availability

All data that were collected where listed in a encrypted and anonymous data base. The dataset is not available but can be requested from the corresponding author.
