# Supplementary material for: Antibiotics prescription and guidelines adherence in elderly: impact of the comorbidities
Source: BMC Geriatr. 2019 Oct 29;19:291. doi: 10.1186/s12877-019-1265-1 (PMC6819552; doi:10.1186/s12877-019-1265-1)
Supplement: Supplementary file 1 — Table S1. Site of infection and antibiotics recommended by the prescriptions guidelines. Table S2. Clinical characteristics of total population: site of infection and Antibiotics prescribed. (DOCX 28 kb) [file 12877_2019_1265_MOESM1_ESM.docx]

Table S1. Site of infection and antibiotics guidelines

| Site of infection | Antibiotics recommended | Duration | References |
| --- | --- | --- | --- |
| Pyelonephritis | Amoxicillin, Amoxicillin/ampicillin and enzyme inhibitor, 3^rd^ generation cephalosporins , Quinolones, Sulfonamides and trimethoprim, | 10-14 days | (14) |
| Cystitis | Amoxicillin, Amoxicillin/ampicillin and enzyme inhibitor, 3^rd^ generation cephalosporins , Quinolones, Sulfonamides and trimethoprim, Nitrofurantoin | 5-7 days |  |
| Male urinary tract infection | Parenteral 3^rd^ generation cephalosporins, Quinolones, Sulfonamides and trimethoprim, | 14-21 days |  |
| Pulmonary tract infection | Amoxicillin, Amoxicillin/ampicillin and enzyme inhibitor, 3^rd^ generation cephalosporins, Quinolones (Levofloxacin), Macrolides | 7-14 days | (15) |
| Exacerbation of Chronic Obstruction Pulmonary Disease | Amox, Amoxicillin/ampicillin and enzyme inhibitor, 3^rd^ generation cephalosporins, Quinolones (Levofloxacin), Macrolides and streptogramins | 7-14 days |  |
| Ski and soft tissu | Amoxicillin / Macrolides and streptogramins / Lincosamides | 10-20 days | (16) |
| *Clostridium difficile* diarrhea | Imidazole derivatives, Glycopeptides (Vancomycin), Fidaxomicin | 10 days | (17) |

Table S2. Clinical characteristics of population : site of infection and antibiotics prescription

|  | | Total  N= 128 (%) | | Antibiotics prescription in accordance with the guidelines  N= 59 (%) | | Antibiotics prescription not in accordance with the guidelines  N= 69 (%) | p-Value * |
| --- | --- | --- | --- | --- | --- | --- | --- |
| **Site of infection** | | | | | | | |
| Pyelonephritis, n (%)  Cystitis, n (%)  Male urinary tract infection, n (%) | 11 (8.6)  37 (28.9)  11 (8.6) | | 6 (10.2)  20 (33.9)  2 (3.4) | | 5 (7.2)  17 (24.6)  9 (13) | | 0.753  0.328  0.63 |
| **Pneumonia, n (%)** | **32 (25)** | | **26 (44.1)** | | **6 (8.7)** | | **p<0.001** |
| Exacerbation of COPD, n (%) | 1 (0.8) | | 0 | | 1 (1.4) | | 1 |
| Uncertain site of infection, n (%) | 32 (24%) | | - | | - | |  |
| ***Clostridium difficile* diarrhea, n (%)** | **4 (3.1)** | | **4 (6.8)** | | **0** | | **0.043** |
| **Antibiotics**  Amoxicillin, n (%)  **Amoxcillin-clavulanic acid, n (%)**  **Third generation cephalosporin, n (%)**  Macrolide, n (%)  Trimethoprim-Sulfamethoxazole-, n (%)  Fluoroquinolone, n (%)  **≥ 2 antibiotics, n (%)**  Others, n (%) | 27 (21.1)  **42 (32.8)**  **36 (28.1)**  1 (0.8)  7 (5.5)  25(19.5)  **17 (13.3)**  12 (9.4) | | 15 (25.4)  **26 (44.1)**  **9 (15.3)**  0  1 (1.7)  8 (13.8)  **3 (5.1)**  4 (6.8) | | 12 (17.4)  **16 (23.2)**  **27 (39.1)**  1 (1.4)  6 (8.7)  17 (24.6)  **14 (20.3)**  8 (11.6) | | 0.285  **0.015**  **0.003**  0.123  0.125  **0.017**  0.383 |
